# Efficacy and cost-effectiveness of a web-based intervention with mobile phone support to treat depressive symptoms in adults with diabetes mellitus type 1 and type 2: design of a randomised controlled trial

**DOI:** 10.1186/1471-244X-13-306

**Published:** 2013-11-15

**Authors:** Stephanie Nobis, Dirk Lehr, David Daniel Ebert, Matthias Berking, Elena Heber, Harald Baumeister, Annette Becker, Frank Snoek, Heleen Riper

**Affiliations:** 1 Division of Online Health Training, Innovation Incubator, Leuphana University Lüneburg, Lüneburg, Germany; 2 Department of Clinical Psychology and Psychotherapy, Philipps-University Marburg, Marburg, Germany; 3 Albert-Ludwigs-University Freiburg, Freiburg, Germany; 4 Department of General Medicine/Family Medicine, Philipps-University Marburg, Marburg, Germany; 5 Department of Medical Psychology, VU University Medical Centre, Amsterdam, The Netherlands; 6 Department of Clinical Psychology, VU University, Amsterdam, The Netherlands; 7 Institute for Health and Care Research (EMGO), VU University Medical Centre, Amsterdam, The Netherlands

**Keywords:** Diabetes, Depression, Guided web-based self-help, Internet, Randomised controlled trial, Efficacy, Cost-effectiveness

## Abstract

**Background:**

A diagnosis of diabetes mellitus types 1 or 2 doubles the odds of a comorbid depressive disorder. The combined diseases have a wide range of adverse outcomes, such as a lower quality of life, poorer diabetes outcomes and increased healthcare utilisation. Diabetes patients with depression can be treated effectively with psychotherapy, but access to psychological care is limited. In this study we will examine the efficacy and cost-effectiveness of a newly developed web-based intervention (GET.ON Mood Enhancer Diabetes) for people with diabetes and comorbid depressive symptoms.

**Methods/Design:**

A two-arm randomised controlled trial will be conducted. Adults with diabetes (type 1 or type 2) with increased depression scores (> 22 on the German version of the Center for Epidemiological Studies Depression Scale (CES-D)) will be included. Eligible participants will be recruited through advertisement in diabetes patient journals and via a large-scale German health insurance company. The participants will be randomly assigned to either a 6-week minimally guided web-based self-help program or an online psychoeducation program on depression. The study will include 260 participants, which will enable us to detect a statistically significant difference with a group effect size of d = 0.35 at a power of 80% and a significance level of p = 0.05. The primary outcome measure will be the level of depression as assessed by the CES-D. The secondary outcome measures will be: diabetes-specific emotional distress, glycaemic control, self-management behaviour and the participants’ satisfaction with the intervention. Online self-assessments will be collected at baseline and after a 2 months period, with additional follow-up measurements 6 and 12 months after randomisation. The data will be analysed on an intention-to-treat basis and per protocol. In addition, we will conduct an economic evaluation from a societal perspective.

**Discussion:**

If this intervention is shown to be cost-effective, it has considerable potential for implementing psychological care for large numbers of people with diabetes and comorbid depression in routine practice and improve health outcomes.

**Trial registration:**

German Clinical Trial Register (DRKS): DRKS00004748.

## Background

Diabetes is highly prevalent. The International Diabetes Federation (IDF) estimates that in 2012, more than 371 million people worldwide were adversely affected by diabetes. It is estimated that 5.2 million people in Germany suffer from diabetes [1]. A meta-analysis found that approximately 11% of all people with diabetes, that were assessed through standardised diagnostic interviews, suffered from depression. However, 31% of all people with diabetes reported themselves as depressive when assessed through self-report questionnaires [2]. In comparison to the general population, a diagnosis of diabetes type 1 or type 2 doubles the odds of a comorbid depressive disorder [2]. The combination of diabetes and depression is associated with poor mental health and harmful medical outcomes. People affected by comorbid depression exhibit lower quality of life [3,4], poorer adherence to diabetes self-care practises [5], worse glycaemic control [6] and greater risk of diabetes-related complications [7]. Furthermore, comorbid depression in diabetes is associated with excess mortality [8,9]. Studies have shown that indirect and direct health care costs are greater among adults with diabetes and depression compared with adults with diabetes only [10-13].

Psychotherapy has been demonstrated to be as effective for people with diabetes and co-morbid depression as for those without diabetes [14,15]. However, depression is poorly recognised in general [16] and depression in diabetes is often undetected and untreated [17]. The reasons are manifold, for example due to the overlap of the symptoms of diabetes and depression, depression is not always diagnosed [18,19], or due to perceived stigmatisation of depression [20]. Using the Internet to provide guided self-help interventions may help to overcome some of the limitations of traditional treatment services. The Internet exhibits anonymity and time-independent access to treatment at low costs [21,22].

Meta-analyses have shown that web-based interventions are effective for treating mental disorders such as depression, anxiety or problem drinking [23-29] and may be successfully implemented in routine practice [30,31]. There are few web-based treatment studies for comorbid disorders, but the results are promising [32,33]. Van Bastelaar et al. (2011) conducted a study to test the efficacy of a web-based diabetes-specific cognitive behavioural therapy (CBT) intervention for persons with diabetes and depression. They showed that the web-based CBT program was efficacious in reducing depressive symptoms in persons with diabetes type 1 or type 2 (effect size d = 0.29) [32]. Moreover, a secondary analysis found that clinical severity was not a significant effect modifier [34]. Thus, web-based interventions may be effective for people with diabetes and comorbid sub-threshold or major depression (according to Diagnostic and Statistical Manual of Mental Disorders (DSM-IV)). This result aligns with the meta-analysis by Andrews and colleagues about the potential of web-based interventions for treating severe mental depression and anxiety [26].

Non-compliance and low adherence to physical treatments or medication regiments are common problems among people with diabetes [35], people with depression [36,37] and participants in web-based interventions in general [38]. As van Bastelaar et al. [32] noted, it is important to consider ways to minimise attrition rates.

After considering the findings of van Bastelaar et al. [32,34,39,40], we developed a minimally guided, diabetes-specific web-based intervention with mobile phone support to reduce depressive symptoms in the German speaking population of diabetes patients. Subsequently, we will determine whether the findings of van Bastelaar et al. [32] can be replicated and whether short-term improvement can be sustained in the long-term. We hypothesise that the intervention group (IG), which will have access to the online program GET.ON Mood Enhancer Diabetes (GET.ON M.D.), will have significantly lower levels of depressed mood than the control group (CG).

Web-based depression interventions appear to be cost-effective for the general population as shown in a small number of studies [41-44]. Although, a web-based diabetes-specific intervention has shown to be effective in reducing depressive symptoms, it is unclear whether it is also cost-effective [32]. Hence, the present trial will evaluate the economic impact of GET.ON M.D.. We hypothesise that from a societal perspective, the GET.ON. M.D. will be a cost-effective program for reducing depressive symptoms of people with diabetes in comparison to the control group.

## Methods/Design

### Study design

This study is designed as a randomised controlled trial with two parallel groups. The IG will receive access to the diabetes-specific, web-based online program (GET.ON M.D.). The CG will receive general online psychoeducation about depression. Moreover, the CG will have the opportunity to work with an unguided version of the online program 12 months after baseline assessment. The participants in both conditions will have unrestricted access to treatment as usual during the study.

**Figure 1:**
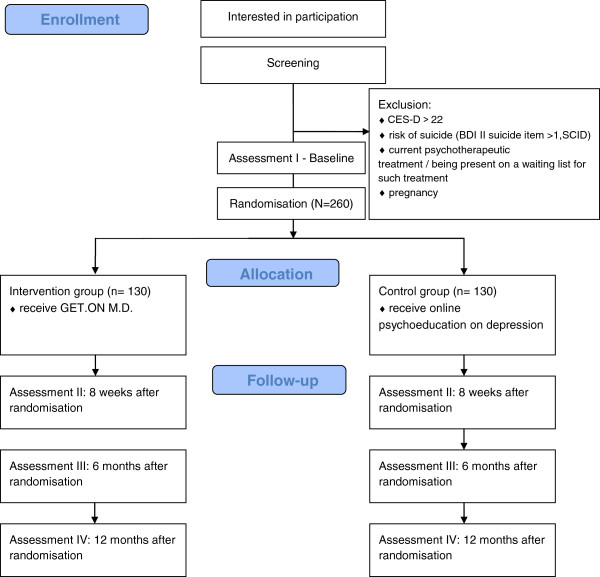
Study flow.

Measurement points are scheduled equally for both groups, at baseline and 8 weeks after randomisation. In addition, there will be follow-up measurements 6 and 12 months after randomisation (Figure 1).

This study was approved by the Psychology Ethics Committee of the Philipps-University of Marburg, Germany.

### Recruitment

Firstly, we will advertise the study in German journals for people with diabetes. Secondly, we cooperate with a large-scale German health insurance company: insurants with diabetes will be informed via post about the possibility of participating in this study. Furthermore, we will contact large-scale diabetes patients organisations with the request to inform their members about our study on their websites. In addition, the study will be advertised on social networks such as Facebook and Twitter.

The recruitment message for the study focuses on the opportunity for people with diabetes mellitus (type 1 or type 2) to help reduce depression. Recruitment is still ongoing (start March 2013) with a total recruitment time of 8 months.

### Study procedures

The open access website (https://geton-training.de) contains detailed information about the study. Interested visitors may leave their email addresses on this website. Afterwards, they will be registered in a secured web-based platform and invited for an online screening. The people who do not pass the screening will receive an explanation for their exclusion along with the German patient guidelines for depression [45] via email. The people who report suicidal ideation will receive an email with the advice to seek professional help and detailed information on available services. The participants who pass the screening will be asked for informed consent via email and will have to fill out a baseline questionnaire. These participants will also be invited to complete the Structured Clinical Interview (SCID-I for DSM IV, by telephone) [46]. The SCID is used to assess whether the included participants have major or minor depression at baseline and whether they have had episodes of depression in the past. The SCID also includes a suicide protocol that enables us to identify people who are currently suffering from suicidal ideation. The telephone SCID is comparable to a face-to-face interview [47] and administered by a trained interviewer. After the telephone interview, the participants will be randomised.

To increase the probability that the participants will complete the follow-up procedure (8 weeks, 6 months and 12 months after baseline), they are offered a monetary incentive for completing each online survey (50 Euros for the completion of all follow-up surveys). Payment will be arranged after each complete questionnaire has been received.

### Study population

This study focuses on adults with diabetes mellitus type 1 or type 2 and depressive symptoms.

#### The following inclusion criteria apply

Aged 18 or above; German language skills; presence of diabetes mellitus type 1 or type 2 diagnosed more than 3 months prior to the study; depressed mood, as indicated by a value of 23 or higher [48,49] on the Center for Epidemiological Studies Depression Scale (CES-D); access to the Internet and an email address.

#### The following exclusion criteria apply

A risk of suicide as measured by item 9 of the Beck Depression Inventory II (BDI II) (value > 1) [50]; a risk of suicide as measured by the SCID [46] and an attached suicide protocol; current psychotherapeutic treatment or being present on a waiting list for such treatment; or pregnancy.

### Sample size

Informed by previous studies [25,32], the sample size is based on the expected difference in the primary outcome (CES-D) between the intervention group and the control group at post-test. This sample size provides 80% power to detect a mean difference of d = 0.35 in the effect sizes for the primary outcome (alpha = 0.05, two-tailed test; calculated using Power Analysis and Sample Size Software (PASS 12)). We will need 130 participants per group; hence a total of 260 participants will be included.

### Randomisation

An automated, web-based randomisation program (randomisation.eu) will assign the participants to either the intervention or the control group in a ratio of 1:1.

### Description of the two groups

#### Intervention group

The GET.ON M.D. program consists of 6 minimally guided online sessions. Each session has an average duration of 45 minutes and includes homework assignments and a mood diary. The participants will be advised to work on one or two sessions each week. Guidance will be provided by graduate students and psychologists (trainers). Trainers are supervised by an experienced clinical psychologist. The communication between the participants and the trainers will take place via the internal messaging function on the GET.ON M.D. platform.

The primary task of the trainer is to reflect on the processed sessions to increase the participants’ motivation and adherence. The trainer will spend approximately three hours in total with each participant. The web-based program is designed to be flexible, and the participants will be able to follow the program on the Internet – also on a tablet or on a mobile phone. An integrated read-aloud function will allow the participants to follow the audio-narration of each session.

### Intervention content

GET.ON M.D. is based on two main evidence-based components: systematic behavioural activation [51] and problem solving [52]. In addition, the program also focuses on relevant diabetes-specific topics, including worries about diabetes complications, physical training, blood glucose, the physician-patient relationship and sexuality [53,54]. The program was developed by consolidating our body of research, clinical experience and input from a diabetes medical specialist (Table 1).

**Table 1:** Overview of the sessions

**Session**	**Depression-specific topics**	**Diabetes-specific topics added**
1	Psychoeducation	Connection diabetes and depression
2	Behavioural activation	Worrying about diabetes-related complications
3	Problem solving I	Diabetes and relationship/sexuality
4	Problem solving II	Rumination about diabetes
5	Behavioural activation, focus on physical activity and self-esteem	Physical activity and diabetes
6	Plan for the future	Conversation with a general practitioner
**Additional components**		
**Session**	**Depression-specific topics**	**Diabetes-specific topics added**
7	Overweight/obesity	Diabetes and nutrition
8	Insomnia	---
**Session**	**Depression-specific topics**	**Diabetes-specific topics added**
Booster	Summary: Behavioural activation; Problem solving	Choice of the known topics (1-6)

### Psychoeducation (session 1)

The first session consists of psychoeducation [55]. The participants are informed that diabetes and depression are linked and can impact each other. In two videos, they are provided psychoeducational information, and they receive an overview of the whole program. After defining their goals concerning depression and diabetes, the participants choose a first positive activity for the next day.

### Behavioural activation (sessions 2 and 5)

The concept of behavioural activation is introduced with a video. The participants are familiarised with the connection between pleasant activities and mood. With the help of examples, the participants create an individual list of activities. Based on the principles of action planning and coping planning, the participants schedule pleasant activities for a few days [51]. Starting with session 2, this scheduling component is integrated into each session.

In session 5, the key focus is on physical activity. Based on the transtheoretical model of health behaviour change [56], the participants can reflect on their own stage of change and receive adapted information and tasks.

### Problem solving (sessions 3 and 4)

Throughout sessions 3 and 4, the main focus is on problem solving [52]. After listing experienced problems, the participants are prompted to classify their problems as solvable or unsolvable. Following a video, the participants become familiar with a six-step plan to handle solvable problems. Next, they are encouraged to apply this plan to one of their problems. In session 4, the participants retrospectively review their plans and choose to either continue to work on the specific problem or start to work on a new one. In addition, they learn techniques to reduce rumination on unsolvable problems.

### Plan for the future (session 6)

In the last session, the participants assess whether they have seen improvements in mood. They can learn about additional ways to get help, if needed. In addition, the participants reflect on which techniques suit them, and are encouraged to make a plan for the future. At the end, they write a letter to themselves about what they want to achieve during the next four weeks.

### Optional sessions and booster session

The participants are offered two optional sessions (healthy sleep and coping with obesity) that are available at the end of sessions 2 through 5. It is known that insomnia is a predictor and a symptom of depression [57]. The sleep session offers information on the principles of sleep hygiene, sleep restriction and stimulus control [58]. There is also a relationship between obesity and depression [59]. In particular, people with diabetes type 2 often suffer from obesity [60]. The aim of this session is to cope with the psychological consequences of obesity, for example frustration, feelings of self-blame and guilt.

Four weeks after finishing the intervention, the participants have the opportunity to work on an optional booster session. The aim of this session is to evaluate progress and to strengthen their newly acquired skills.

### Improving efficacy and adherence

Taking into account that non-compliance presents as a problem in web-based programs [38], in many depressed people [36,37] and people with diabetes [35], we developed two ways to increase the number of sessions completed by the intervention group.

### Improving efficacy and adherence I: text message coach

Preliminary results have shown that mobile phone-based interventions and mobile phone supportive messages have the potential to improve psychological wellbeing and decrease stress or depression [61]. A pilot study demonstrated that daily text messaging, as a supplement to face-to-face CBT, is feasible and acceptable to patients. Positive experiences with the text messages were reported by 65% of the participants [62]. Studies have also indicated that text messages about diabetes self-management can also lead to benefits in some cases [63,64]. The participants in this study will have the opportunity to apply for a standardised text message coach: they will receive daily text messages to support their efforts to integrate the techniques they learn within the program into everyday life. The text messages address four different areas: behavioural activation, problem solving, diabetes and motivation enhancement.

### Improving efficacy and adherence II: phone call

Clarke et al. (2005) have demonstrated that reminders (by telephone or postcards) are a pivotal factor in effectiveness and attrition [65]. In the GET.ON M.D. study, a participant who does not complete a session within one week will receive a reminder email from his or her personal trainer. If a participant does not log in to the program during the next seven days after receiving this reminder, he or she will receive a phone call from his or her trainer.

The aim of this call is both to motivate the participant to proceed with the training and to identify the exact reasons for not logging in (e.g. forgetfulness, technical problems, lack of interest).

### Control group - psychoeducation

The CG will have access to online psychoeducation based on the German S3-Guideline/National Disease Management Guideline “Unipolar Depression” [45]. Psychoeducation is helpful in reducing depressive symptoms [55]. It informs the participants about the nature of depression including information about symptoms and sources of help. After the last measurement point (12 months after baseline), the CG will have access to an unguided version of the GET.ON M.D. program.

### Outcome assessment

For an overview of the assessments at baseline, after treatment and during the follow-up phase see Table 2.

**Table 2:** Overview of the assessments

	**T0**	**T1**	**T2**	**T3**	**T4**
**Measurements for the efficacy evaluation**					
Center for epidemiological studies depression Scale	**x**	**x**	**x**	**x**	**x**
Hospital anxiety and depression scale (Depression)	**-**	**x**	**x**	**x**	**x**
Penn state worry questionnaire	**-**	**x**	**x**	**x**	**x**
Behavioural activation depression scale	**-**	**x**	**x**	**x**	**x**
Social problem solving inventory - Revised	**-**	**x**	**x**	**x**	**x**
Haemoglobin A1c	**-**	**x**		**x**	**x**
Problem areas in diabetes	**-**	**x**	**x**	**x**	**x**
Acceptance and action diabetes questionnaire	**-**	**x**	**x**	**x**	**x**
Diabetes self-management questionnaire	**-**	**x**	**x**	**x**	**x**
Kessler psychological distress scale	**-**	**x**	**-**	**-**	**-**
Negative effects of online health trainings	**-**	**-**	**(x)**	**-**	**-**
Attitudes towards seeking psychological help scale	**-**	**x**	**x**	**-**	**-**
**Measurements for the economic evaluation**					
Trimbos/iMTA questionnaire for costs associated with Psychiatric Illness	**-**	**x**	**-**	**x**	**x**
Quality of life (EuroQol, SF-12)	**-**	**x**	**x**	**x**	**x**
**Additional measurements**					
Demographic variables questionnaire	**x**	**-**	**-**	**-**	**-**
Lifestyle data	**x**	**-**	**-**	**-**	**-**
Data on diabetes	**x**	**-**	**-**	**-**	**-**
Patient questionnaire on therapy expectation and evaluation (Online-Training)	**-**	**x**	**-**	**-**	**-**
Client Satisfactory Questionnaire	**-**	**-**	**(x)**	**-**	**-**
Way of recruitment and familiarity with the Internet	**x**	**-**	**-**	**-**	**-**

*T0* Screening*, T1* Baseline*, T2* 8 weeks*, T3* 6 months*, T4* 12 months*.*

*Assessments: x =* intervention and control group*, (x) =* intervention group only*.*

### Primary outcome measures

The primary outcome is the level of depression, which is measured by the CES-D, a well-validated self-report depression questionnaire that will be administered online [48,49]. The CES-D consists of 20 items that are answered on a 4-point Likert scale; the total score ranges between 0 and 60. The internal consistency is reported to be α = 0.89, and the split-half reliability is r = 0.91 [48].

### Secondary outcome measures

The secondary outcome measures include the depression subscale of the Hospital Anxiety and Depression Scale (HADS [66,67]); the Penn State Worry Questionnaire (PSWQ [68], 3-item short version [69]); the Behavioural Activation Depression Scale (BADS [70], 9-item short version [71]); the Social Problem Solving Inventory-Revised (SPSI-R [72]); the long-term blood sugar level (self-reported haemoglobin A1c values (HbA1c)); the Problem Areas in Diabetes questionnaire (PAID [73], German 5-item short version [74]); the Acceptance and Action Diabetes Questionnaire (AADQ [75], German version [76]); the Diabetes Self-Management Questionnaire (DSMQ [77]); the negative effects of online health trainings questionnaire ([78], self-adapted version for online health trainings); the Kessler Psychological Distress Scale (K10 [79]); and the Attitudes Towards Seeking Psychological Help Scale (ATSPHS, [80], 10-item short version [81]).

### Additional measurements

We will collect socio-demographic data (age, gender, education and occupation); lifestyle data (body mass index, smoking and alcohol use); data on diabetes (type, duration of illness and comorbidity); Patient Questionnaire on Therapy Expectation and Evaluation (PATHEV [82]); data on satisfaction with the program (a self-designed questionnaire based on the German version of the Client Satisfaction Questionnaire [83,84]); data about the method of recruitment (for example through a diabetes journal or Facebook) and additional information about the participant’s familiarity with the Internet.

### Measurements for the economic evaluation

#### Cost measures

We will use the Dutch Cost Questionnaire “Trimbos Institute and Institute of Medical Technology Questionnaire for Costs Associated with Psychiatric Illness” (TiC-P) [85] adapted for the German health care system. We will include the costs of any type of health care and medicines (direct medical costs), travel expenses and also those for parking (direct non-medical costs), and the costs related to production losses (indirect costs) [86].

### Quality of life

Quality-adjusted life years (QALYs) will be calculated based on the EuroQol (EQ-5D) [87] and the Short Form Health Survey 12 (SF-12) [88]. The EQ-5D is a validated tool for measuring general health-related quality of life. It consists of five items (mobility, self-care, usual activities, pain/discomfort and anxiety/depression), each of which is rated as causing “no problems”, “some problems” or “extreme problems” [89]. Moreover, to generate the QALYs, a visual analogue scale (VAS) ranging from 0 (worst imaginable health state) to 100 (best imaginable health state) is also an integral part of the EQ-5D.

The SF-12 is based on the 36-item Short Form Health Survey (SF-36), a health-related quality-of-life questionnaire [90]. The SF-12 focuses on eight dimensions of health (physical functioning, role-physical, bodily pain, general health, vitality, social functioning, role-emotional and mental health).

### Statistical analysis

#### Clinical analysis

The analysis will be performed according to the Consolidated Standards of Reporting Trials (CONSORT) statement regarding eHealth [91]. The data will be analysed on an intention-to-treat (ITT) basis. Group differences in the baseline values of the primary outcome will be compared using t tests to assess whether randomisation was successful. Missing data will be addressed following the recommendations of Little and Rubin [92] and Schafer [93]. We will analyse the CES-D data at two months post-treatment using between-group analyses of covariance (ANCOVAs) on the individual baseline depression scores, adjusting for sex, age and socio-economic status. We will use Cohen’s d to measure the between-group effect size. Cohen’s d will be calculated as the difference between the mean post-test scores of the intervention group and the control group divided by the pooled standard deviation [94]. All of the other secondary outcomes will be analysed in the similar way.

We will also conduct clinical significance change analyses as described by Jacobson and Truax [95,96]. In the first step, we will test whether the changes from pre-test to post-test are statistically reliable and build a reliable change index (RCI). In the second step, we will calculate the clinical significance. Based on the reliable change index, the participants who display a reliable positive change, no change or a reliable deteriorated change will be classified as responders, non-responders or deteriorated, respectively [95].

As secondary analyses, we will also conduct per-protocol (PP) and completers-only analyses.

To show the long-term effect on the primary outcome after 6 months and 12 months, we will use mixed models.

### Economic evaluation

The economic analysis will be performed from a societal perspective. We will conduct a cost-effectiveness analysis (CEA) and a cost-utility analysis (CUA). Using the incremental cost-effectiveness ratio (ICER), we will show the differences in the costs and benefits between the two groups. The robustness of the ICER will be determined using non-parametric bootstrapping. Furthermore, we will also use bootstrapping to quantify the uncertainty around the ICER, which will be shown on the cost-effectiveness plane [97] and as a cost-effectiveness acceptability curve [98,99]. To perform the CUA, we calculate QALYs from the EQ-5Q and also from the SF-12.

## Discussion

In the present study, we aim to compare a web-based program with mobile phone support and minimal guidance with a control group, receiving psychoeducation only. We expect the depression level of the participants in the intervention group to be significantly lower immediately after the intervention and also after 6 and 12 months compared with the control group. Our planned analyses will enable us to assess whether the outcomes are sustained over time.

Depressive symptoms in people with diabetes have an adverse impact on mental health, quality of life and on medical outcomes such as blood glucose [3-7]. Compared to the comprehensive body of evidence concerning web-based interventions for depression, very little is known about the efficacy of web-based interventions in persons with comorbid (somatic) disorders. This study will contribute to the evaluation of evidence concerning web-based interventions for participants with diabetes and co-morbid depression.

Depression treatments for adults with diabetes produce economic benefits [100]. Until now, there has been no evidence about the cost-effectiveness of web-based treatments for diabetes with comorbid depression. We will conduct CEA und CUA from a societal perspective. If the intervention is cost-effective, these findings may encourage the health care system (including health insurance companies) to integrate those web-based programs into routine diabetes care.

Because adherence to recommendations involving treatment recommendations and lifestyle changes is often a problem with diabetes patients [35], depressive people [36,37] and with participants in web-based interventions [38], we will use two methods to improve the efficacy of the intervention by increasing the number of sessions completed by the intervention group. Firstly, all of the participants can receive daily text message support [61-64]. Secondly, all of the participants who have not logged in to the program seven days after an email reminder will be contacted by their trainer by phone [65]. The trainer will then try to motivate the participant to continue with the intervention. We decided to focus on these two methods; however there are other approaches such as the concept of persuasive technology [101]. It is unknown which methods should be recommended, so further studies should examine which ones are effective concerning web-based interventions.

### Limitations

One limitation of this study design is that many diabetes patients are elderly, especially those with diabetes type 2 and may have limited access to the Internet. In 2002, only 8% of the Germans 60 or older had access to the Internet: this number increased to 39% ten years later [102]. The results from this study cannot be generalised to the overall population of patients with diabetes (especially type 2) and comorbid depression.

Furthermore, it is known that lower social status and lower education are associated with an increased risk for diabetes [103] and that women (28%) have significantly higher rates of depression compared to men (18%) [2]. As studies show, web-based interventions are mostly used by higher educated persons and females [32,104,105]. Therefore, we expect selection bias, restricting the external validity, particularly with regard to lower educated and males with diabetes.

## Conclusions

Given the high prevalence of comorbid diabetes and depression, web-based interventions may have great potential to lower the threshold and increase access to the treatment of these co-morbid disorders. If GET.ON M.D. is shown to be cost- effective, it has great potential to be integrated into routine care and so to improve the well-being of people with diabetes and comorbid depression. This is of high importance given the negative impact of co-occurrence of diabetes and depression on psychological and medical outcomes, as well as on the health care system [3-7,10,12,14,100].

Further research is necessary to establish whether interventions that integrate diabetes and depression are more effective in the long term compared with conventional depression interventions.

## Abbreviations

AADQ: Acceptance and action diabetes questionnaire; ANCOVAs: Analyses of covariance; ATSPHS: Attitudes toward seeking psychological help scale; BADS: Behavioural activation depression scale; BDI II: Beck depression inventory II; CBT: Cognitive behavioural therapy; CEA: Cost-effectiveness analysis; CES-D: Center for epidemiological studies depression scale; CG: Control group; CONSORT: Consolidated standards for reporting trials; CUA: Cost-utility analysis; DRKS: Deutsches register für klinische studien (German register for clinical trials); DSM-IV: Diagnostic and statistical manual of mental disorders IV; DSMQ: Diabetes self-management questionnaire; EQ-5D: EuroQol; GET.ON M.D.: GET.ON mood enhancer diabetes; HADS: Hospital anxiety and depression scale; HbA1c: Haemoglobin A1c; ICER: Incremental cost-effectiveness ratio; IDF: International diabetes federation; IG: Intervention group; ITT: Intention-to-treat; K10: Kessler psychological distress scale; PAID: Problem areas in diabetes; PASS 12: Power analysis and sample size software; PP: Per-protocol; PSWQ: Penn state worry questionnaire; PATHEV: Patient questionnaire on therapy expectation and evaluation; QALYS: Quality adjusted life years; RCI: reliable change index; SCID: Structured clinical interview for DSM IV; SF-12: Short form health survey 12; SF-36: Short form health survey 36; SPSI-R: Social problem solving inventory-revised; TIC-P: Trimbos institute and institute of medical technology questionnaire for costs associated with psychiatric illness; VAS: Visual analogue scale.

## Competing interests

Professor Berking is minority shareholder of Minddistrict GmbH which provides the online platform for the training.

## Authors’ contributions

MB obtained funding for this study. All authors contributed to the design of the study. SN, DL, DDE, MB, EH, FS and HR developed the intervention content. HB and AB contributed to the intervention content. SN wrote the draft of the manuscript. HR supervised the writing process. All authors contributed to the further writing of the manuscript and approved the final manuscript.

## Pre-publication history

The pre-publication history for this paper can be accessed here:

http://www.biomedcentral.com/1471-244X/13/306/prepub
